# *Notes from the Field:* Four Cases of Lyme Disease at an Outdoor Wilderness Camp — North Carolina, 2017 and 2019

**DOI:** 10.15585/mmwr.mm6904a5

**Published:** 2020-01-31

**Authors:** Alexis M. Barbarin, Steven W. Seagle, Susan Creede

**Affiliations:** ^1^Communicable Disease Branch, North Carolina Division of Public Health, North Carolina Department of Health and Human Services, Raleigh, North Carolina; ^2^Southern Appalachian Environmental Research and Education Center and Department of Biology, Appalachian State University, Boone, North Carolina; ^3^Buncombe County Health and Human Services, Asheville, North Carolina.

On June 10, 2019, the North Carolina Division of Public Health was notified by the Buncombe County Health Department of three cases of Lyme disease among children aged 6–8 years. Lyme disease is a bacterial infection transmitted by the bite of an *Ixodes scapularis* tick that is infected most commonly with the bacterium *Borrelia burgdorferi*. An investigation conducted by Buncombe County communicable disease nurses determined that all three children were homeschooled and had attended a local, year-round, outdoor wilderness day camp. Each child had attended the camp at least 1 day a week over the course of the previous fall and spring. The camp site for the wilderness program is completely outdoors, with a canopy of hardwood forest providing much of the shelter. Further investigation identified an earlier camp participant who had received a diagnosis of Lyme disease in 2017 (Table).

**TABLE:** Demographic information and clinical and laboratory evidence of Lyme disease in four attendees at a wilderness day camp — North Carolina, 2017 and 2019

Patient	Age (yrs)	Sex	Date of illness onset	Clinical evidence	Laboratory evidence	Tick exposure	Treatment	Case classification
**A**	8	Female	May 12, 2019	Brief, recurrent attacks of joint swelling	Positive *Borrelia burgdorferi* IgG western blot	Attended wilderness day camp	Doxycycline	Confirmed
				Arthralgia	Positive *B. burgdorferi* IgM western blot	Ticks removed		
				Physician diagnosis of Lyme disease				
**B**	6	Female	May 1, 2019	Erythema migrans rash	Negative *B. burgdorferi* IgG western blot	Attended wilderness day camp	Doxycycline	Confirmed
				Fever	Positive *B. burgdorferi* IgM western blot	Ticks removed		
				Headaches	Positive Lyme disease antibody EIA, 1.77			
				Arthralgia				
				Loss of appetite				
				Increased fatigue				
				Physician diagnosis of Lyme disease				
**C**	7	Male	May 17, 2019	Small erythematous rash	Negative *B. burgdorferi* IgG western blot	Attended wilderness day camp	Doxycycline	Probable*
				Fever	Positive *B. burgdorferi* IgM western blot	Ticks removed		
				Headaches	Positive Lyme disease antibody EIA, 3.26			
				Physician diagnosis of Lyme disease				
**D**	9	Male	September 28, 2017	Erythema migrans rash	Positive *B. burgdorferi* IgG western blot	Attended wilderness day camp	Doxycycline	Confirmed
				Radiculoneuropathy	Positive *B. burgdorferi* IgM western blot			
				Cranial neuritis (Bell’s palsy)	Positive Lyme disease antibody EIA, 11.08			
				Arthralgia				
				Physician diagnosis of Lyme disease				

**Abbreviations:** EIA = enzyme immunoassay; Ig = immunoglobulin.

* https://wwwn.cdc.gov/nndss/conditions/lyme-disease/case-definition/2017/.

North Carolina has historically had a low incidence of reported Lyme disease cases (*1*) but remains the southernmost border of the leading edge of Lyme disease in the United States (*2*). In North Carolina in 2017, 0.69 confirmed cases of Lyme disease per 100,000 residents were reported, a rate significantly lower than the 2017 national average of 9.1 confirmed cases per 100,000 residents (*3*).

On June 13, a North Carolina interagency assessment team traveled to the wilderness day camp to conduct entomologic surveillance for *Ixodes* ticks. Participants covered a total of 0.27 acres (1,077 m^2^) of land while “flagging and dragging.”* A total of 39 nymphal ticks were collected. Ticks were preserved in 95% ethanol and sent to CDC’s Division of Vector-Borne Diseases in Fort Collins, Colorado, for pathogen testing. Of the 39 ticks collected, 37 (95%) were confirmed as *Ixodes scapularis* ticks by molecular testing. Two ticks yielded poor DNA, and pathogen tests were ruled inconclusive. Six of the 35 ticks yielding DNA suitable for analysis tested positive for *B. burgdorferi* sensu stricto, the causative agent of Lyme disease. One of the six ticks was coinfected with *Borrelia miyamotoi*. All 35 ticks tested negative for *Anaplamsa phagocytophilum* and *Babesia microti* (two pathogens tested for when conducting *Ixodes* tick testing). Results indicated that nymphal ticks collected at the camp site had a *B. burgdorferi* infection prevalence of 17% (95% confidence interval = 8.1–32.7).

This was the first reported cluster of Lyme disease patients with a common exposure to be identified in North Carolina and the furthest south that *Borrelia*-infected ticks have been identified through North Carolina Division of Public Health entomologic surveillance efforts. Clinicians should be aware of the risk for transmission of Lyme disease in North Carolina and consider recommended diagnostic testing and treatment (*4*). To prevent exposure to *Borrelia* and other tickborne diseases, North Carolina Division of Public Health encourages everyone to wear personal protective clothing, to use EPA-approved repellents such as diethyltoluamide (DEET), and to conduct full-body examinations for ticks following outdoor activities in possible tick habitats. Prevention is the best defense against Lyme disease. Close collaboration between the North Carolina Division of Public Health and county health departments, along with clinician awareness, are essential for rapid identification of vector-borne disease expansion and appropriate treatment.

## Footnotes

* To sample the environment for ticks trying to locate a host, light-colored cloths with a wooden leading frame are dragged through grass or a leafy forest floor (dragging), and light colored cloths are used to brush uneven surfaces such as small bushes in wooded areas (flagging).
